# Middle ear neuroendocrine tumor with multiple brain metastases: a case report and literature review

**DOI:** 10.3389/fonc.2024.1392610

**Published:** 2024-05-30

**Authors:** Yesheng Sun, Ying Zhang, Dongpeng Cai, Wei Zhang, Zhiqian Yang

**Affiliations:** ^1^ Department of Neurosurgery, The First Affiliated Hospital of Guangdong Pharmaceutical University, Guangzhou, China; ^2^ Guangdong Provincial Engineering and Technology Research Center of Stem Cell Therapy for Pituitary Disease, Guangzhou, China

**Keywords:** middle ear neuroendocrine tumor, MeNET, carcinoid, brain metastases, operation, radiotherapy, chemotherapy

## Abstract

Middle ear neuroendocrine tumor (MeNET) is a low-grade tumor with rare recurrence or metastasis. Here, we describe the case of a 29-year-old man who suffered from MeNET that recurred 3 times over 10 years and eventually metastasized to the brain. The patient was treated with surgical resection, radiotherapy, and chemotherapy. However, the tumor was not entirely removed as the brain metastatic tumor adhered tightly to the brainstem. Due to tumor rupture and bleeding after multiple brain tumor removal, profound coma developed. Finally, the patient died 10 months after the last surgery. To our knowledge, this is the first report of a MeNET case with multiple brain metastases. Characteristics of the present case indicate that CK, SYN, increased Ki67 index, and ATRX may be potential biomarkers of invasive MeNET. The survival of patients with brain metastatic MeNET may be extended by surgical resection, radiotherapy, and chemotherapy. Close follow-up of distinctive metastases and biomarkers related to recurrence is also suggested.

## Introduction

The diagnosis of tumors with solid trabecular and glandular structures located in the middle ear has been variously described as middle ear adenoma, unilateral adenoma, middle ear benign adenocarcinoma, or adenocarcinoma. A naming controversy remains. Earlier, some researchers insisted that neuroendocrine tumors (carcinoid tumors) and middle ear adenomas were distinct, because middle ear gland tumors can show both neuroendocrine and epithelial differentiation. Therefore, terms in the literature include amphicrine, middle ear carcinoid tumor, middle ear adenomatous tumor, and adeno-carcinoid tumor. All these tumors are composed of at least two distinct cell types, exocrine and neuroendocrine, and in 2005 the World Health Organization (WHO) classified middle ear carcinoid and middle ear adenoma as the same tumor.

However, according to Xu et al (1). the terms “middle ear carcinoid” and “middle ear adenoma” in the WHO classification are not biologically correct. Rather, they recommended that these should be referred to as mixed epithelial and neuroendocrine tumors of the middle ear. Accordingly, the 2017 WHO classification considered these tumors as adenomas with neuroendocrine features (2). Furthermore, a recent study identified 20 patients diagnosed with middle ear adenoma, middle ear carcinoid, or middle ear neuroendocrine adenoma/tumor. Consistent immunohistochemical profile that was positive for SATB2, INSM1, synaptophysin, keratins (AE1/AE3, CAM5.2) and focal CDX2 with occasional weak GATA3was found in these patients, supporting that middle ear adenoma, middle ear carcinoid, or middle ear neuroendocrine adenoma/tumor are the same tumor (3). The 2022 WHO classification of Endocrine and Neuroendocrine Tumors now considers these tumors neuroendocrine neoplasm of the middle ear (4), here designated MeNET (middle ear neuroendocrine tumor).

Neuroendocrine neoplasia is commonly found in the digestive and respiratory systems (73.7% and 25.1% of cases, respectively), but constitutes fewer than 2% of ear tumors (5, 6). Since its first description in 1976 (7), 198 cases have been reported (8) with only 9 cases of distant metastasis. The present report is the first to describe MeNET with multiple recurrences and brain metastases.

## Case presentation

**Figure 1:**
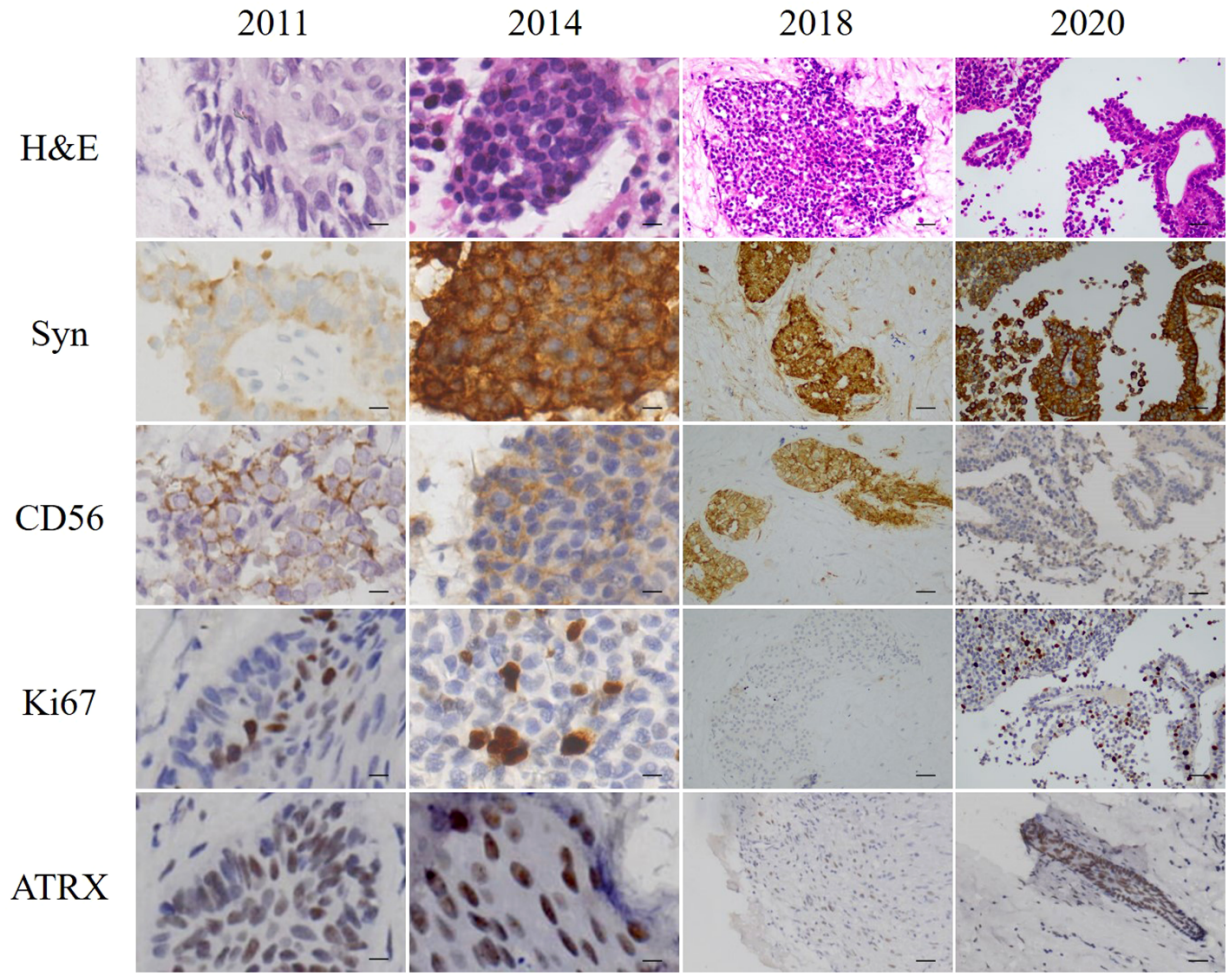
H&E staining and immunohistochemistry of excised tumors. Scale bars for 2011 and 2014 = 10 µm. Scale bars for 2018 and 2020 = 50 µm.

A 29-year-old man was admitted to hospital in September 2011 with right-sided hearing loss, right-sided facial numbness, and dropped corner of the mouth on the left side. A mass in the right external auditory canal was found by computed tomography (CT) scan. A preliminary biopsy suggested neuroendocrine carcinoma. Tumor excision of the right external auditory canal was performed. The tumor showed clear boundaries in the intraoperative opened processus mastoideus area. The incus and malleus were all surrounded by the tumor. Large numbers of tumor was found in the tympanic antrum and tympanic cavity. Hematoxylin-eosin staining (H&E) of the excised tumor showed clumped and tabled cell distribution and some glandular tube-like arrangements. Immunohistochemistry of the excisions displayed typical characteristics of neuroendocrine tumors that were positive for synaptophysin (Syn), CD56, Ki67 (5%), and P63; and negative for chromogranin A (CgA), neuron-specific enolase (NSE), and carcinoembryonic antigen (M-CEA). The nuclei of these cells showed mild atypia; no mitotic figures or necrosis was identified (**Figure 1**). Considering all the above pathological results, the patient was given a diagnosis of MeNET.

**Figure 2:**
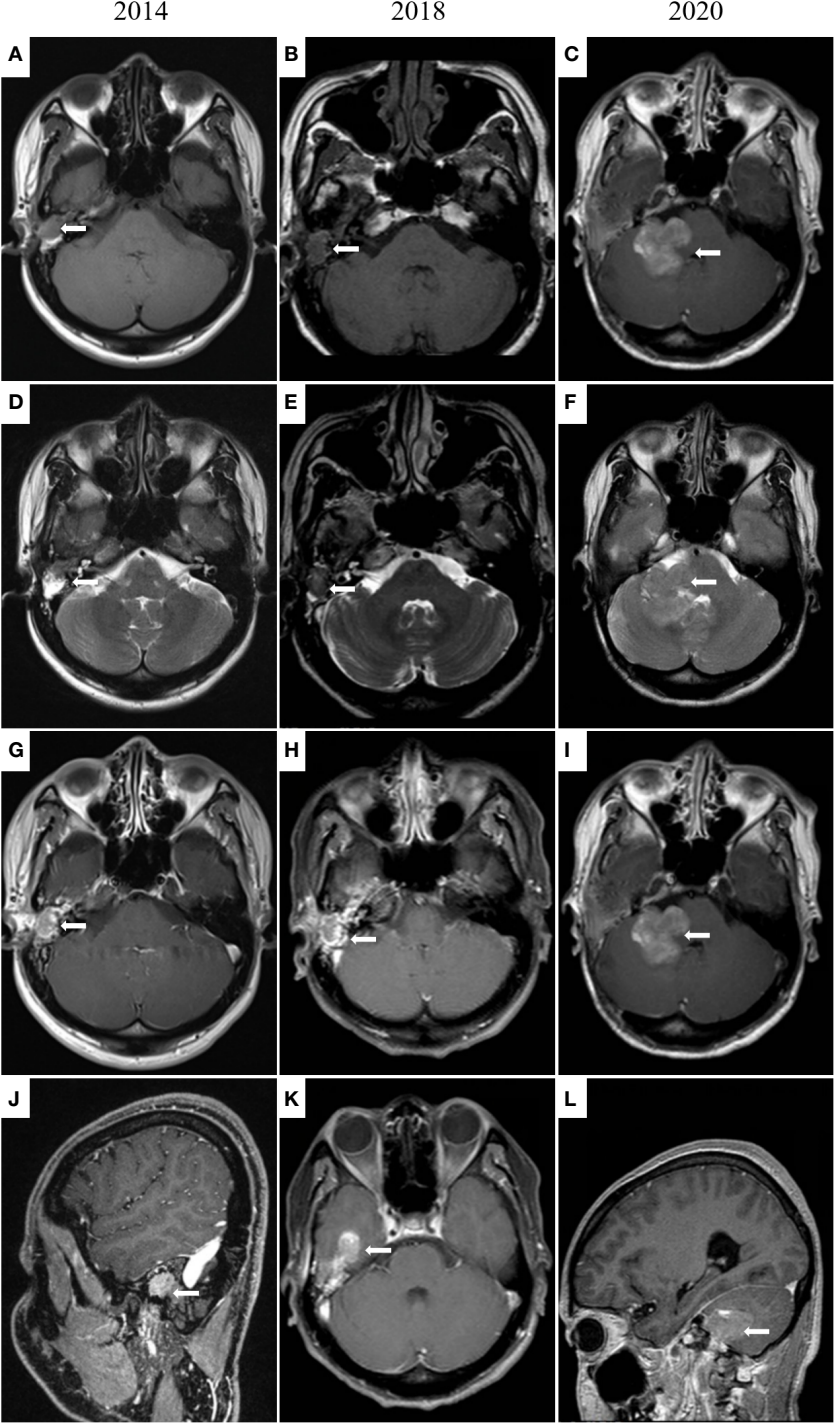
MRI images of the middle ear and brain. In 2014 **(A, D, G, J)**, irregular soft tissue signal shadows in the right external auditory canal, middle ear cavity, and mastoid cells are shown. In 2018 **(B, E, H, K)**, an irregular mass (14 × 18 × 12 mm) in the right external auditory canal and middle ear cavity and tumor invasion into the brain is presented. In 2020 **(C, F, I, L)**, tumor metastases in the right cerebellopontine angle and right temporal meningeal.

In June 2014, the patient revisited our hospital with the right external auditory canal plugged by a smooth mass. Magnetic resonance imaging (MRI) showed tumors and bleeding in the right external ear canal, middle ear cavity, and mastoid cells (**Figure 2**). Tumor resection of the right temporal bone and facial nerve decompression surgery were performed. H&E staining and immunohistochemistry were conducted on the excised tumor. Small groups of gland cell-like clusters with abundant cytoplasm, acidophilia, necrosis, and partial mitotic figures were observed. Positivity of Syn, CD56, Ki-67 (8%), and CK were also found (**Figure 1**), while S-100, CK5/6, and CK7 were each negative. All the pathological features were consistent with neuroendocrine tumors. Hence, the patient was given a diagnosis of MeNET recurrence.

Four months later, the patient presented with multiple swollen lymph nodes in the bilateral neck areas I, II, and III. Postoperative radiotherapy was administered from December 2014 to February 2015. MRI showed shrunken lymph nodes (from 12 × 19 mm to 5 × 13 mm). Unlike the MRI results in December 2014, a ring-enhanced lesion on the posterior edge of the right mastoid was found, and distant metastasis of the tumor was suspected. The patient refused surgical intervention at that time.

Three years later, the patient was referred to our hospital for hearing loss and right ear ache. Tumor invasion of the right temporal lobe and meninges was found (**Figure 2**). During surgery, invasion into the dura and the temporal lobe were confirmed. H&E staining on the excised tumor showed cells of uniform size with round nuclei, rare mitotic figures, and extensive necrosis. There was a small number of abnormally shaped cells arranged in irregular adenoids and patches. Via immunohistochemistry Syn, CD56, Ki-67 (about 5%), CK, and P63 were detected, while results for CK20 and GFAP were negative (**Figure 1**). Therefore, the diagnosis of metastatic MeNET was confirmed.

Two years later, the patient consulted again in hospital with dizziness and right earache. MRI examination suggested new tumor metastases in the right cerebellopontine angle and right temporal meningeal as well as residual tumor in the operative region (**Figure 2**). Resection of the right cerebellopontine angle tumor was performed. Intraoperative exploration of the tumor showed abundant blood supply and tumor adhesion to the cerebellar cortex, brainstem, and dura mater. Due to tight adherence to the brainstem, the capsule of the tumor was not entirely peeled off. Immunohistochemistry of Syn, CD56, Ki-67 (about 10%), CK, and P63 were all positive on the excised tissue (**Figure 1**), but CgA, CK7, TTF1, P40, CDX2 and CK20 were not discovered. The nuclei of these cells showed mild atypia with no evidence of mitotic figures. The histopathological diagnosis was the same as before.

Chemotherapy was administered (combined cisplatin, temozolomide, and anlotinib). Due to tumor rupture and bleeding after multiple brain tumor removal, profound coma developed, and the patient died 10 months after the last surgery.

## Discussion

MeNET, derived from primitive precursor cells or neural crest cells with epithelial and neuroendocrine differentiation, has long been regarded as having indolent malignancy, low risk of invasiveness, and rare distant metastasis. However, a series study of MeNET showed a local recurrence rate of 12.7 to 22%, and a local or distant metastasis rate of 4 to 9% (9, 10).

The biological phenotypes of primary and invasive MeNET are distinct. Primary MeNET is generally observed as small, slow-growing, non-invasive, and with a good prognosis. Tympanomastoidectomy or radical mastoidectomy is the optimal treatment for primary MeNET (11), but the value of adjuvant radiation therapy in reducing local recurrence rates is unclear and controversial. A systematic review reported that patients who received local adjuvant radiotherapy failed to experience a lower recurrence rate compared with patients given surgery alone (8).

To date, 198 MeNET cases have been reported, including only 9 cases of distant metastasis. The invasive metastases were found in the parotid glands, bones, cervical lymph nodes, and liver (**Table 1**). Since reports of distant metastasis have been rare, there is no standardized treatment plan. Generally, patients with cervical lymph node metastasis are advised to receive selective neck dissection or superficial parotidectomy. Some patients with invasive MeNET have been treated with radiotherapy, with doses ranging from 54 to 70 Gy (14, 16). Furthermore, adjuvant radiation therapy is controversial in terms of preventing tumor recurrence; radiation exposure may result in secondary malignant transformations (19).

**Table 1:** Regional or distant metastasis of 10 patients with invasive MeNET.

	Age, y	Sex	TDM	Biomarkers, +	Necrosis	Mitoses	MF	Site of mets	Treatment	Follow-up
This case	29	M	7 y	Syn, CD56, Ki-67 (about 5%), CK, P63	Yes	Yes	NS	R temporal lobe, cerebellopontine angle, meninges	Surgery + RT + CTX	Died
Pt 1 (12)	55	M	8 y	Cg A, vimentin, CKC	Yes	Yes	NS	R-sided cervical lymph node	MRND + RT + CTX	Free for 5 mo
Pt 2 (13)	51	M	20 y	CKC, Syn, chromogranin, polyclonal CEA, pancreatic polypeptide	None	None	None	R parotid gland, parotid lymph node	SPE + SND + RT	Recurrence in the R middle cranial fossa.
Pt 3 (10)	72	M	13 y	Syn, NSE, keratin, synaptophysin	NS	NS	NS	R parotid gland	SPE	Free for 24 mo
Pt 4 (10)	72	F	43 y	Syn, NSE, Cg A, cytokeratin	NS	NS	NS	L parotid lymph node	SPE	Free for 48 mo
Pt 5 (14)	55	F	3 y	NSE, keratin, vimentin, chromogranin	None	None	None	Cervical level I-V lymph node	MRND + RT	Unknown
Pt 6 (15)	45	M	6 y	Syn, CD56, chromogranin, Ki67 (<20%)	None	None	None	Lymphatic and liver metastasis	RT+ CTX	Died
Pt 7 (16)	72	F	8 mo	Cytokeratin AE1-3 and vimentin, chromogranin A, Ki67 (<3%)	None	None	None	L parotid and cervical level II-IV lymph node	SPE + SND + RT	Recurrence after 6 mo
Pt 8 (17)	52	M	11 y	Syn, CD56, keratin, chromogranin	None	None	None	Cervical lymph nodes and iliac crest	SND	Unknown
Pt 9 (18)	58	F	6 y	Syn, pan-cytokeratin, chromogranin, Ki67 (5%)	None	None	None	Liver	Surgery+ RT	Died

CKC, cytokeratin cocktail; F, female; IMRT, intensity modulated radiation therapy; L, left; mets, metastasis; MF, mitotic figure; MRND, modified radical neck dissection; NS, not specified; R, right; M, male; RT, radiotherapy; CTX, Chemotherapy; SND, selective neck dissection; SPE, superficial parotidectomy; TDM, time between the first diagnosis and the metastasis.

Although the therapeutic effects have been limited, patients have also received chemotherapy, including carboplatin and VP16 (15). In the present case, radiotherapy was recommended after relapse, but the patient refused. A multiple resection was performed to remove the brain metastatic tumors, but profound coma developed due to tumor rupture and bleeding. Combined cisplatin, temozolomide, and anlotinib seemed to slow progression of the tumor and improve the patient’s health conditions, but did not change the ultimate outcome.

There are no evidence-based guidelines for MeNET. In the event of recurrent MeNET, the application of radiation therapy may be extrapolated from the recommendations of the NCCN (National Comprehensive Cancer Network) for neuroendocrine tumors of the thymus (category 2B, category 3 with addition of chemotherapy) (15, 20). Currently, the efficacy of radiotherapy and chemotherapy in MeNET is unclear and should be investigated systematically.

Compared with local MeNET, invasive MeNET is a more complicated malady with a much poorer prognosis. The lack of specific biomarkers to differentiate invasive from local MeNET greatly hinders early detection. In the present case, the primary and invasive MeNET were both positive for CgA, NSE, Syn, CD56, cytokeratin, and vimentin, with the highest rates shown by CgA and NSE (42 and 39%, respectively). Of the 9 cases of invasive MeNET reported previously, 8 and 7 (88.9 and 77.8%) showed positive reactions for CK and Syn (**Table 1**). Pellini et al (14). reported an isolated case and detected CK in the neck lymph node but not in the primary MeNET. In the current case, both the primary and metastasized tissues were positive for CK and Syn, suggesting that these may be potential biomarkers for invasive MeNET biomarkers.

Ki-67, a nuclear antigen linked to proliferation, is closely associated with tumor cell growth. Generally, lower Ki-67 values indicate a better prognosis and longer survival, with less likelihood of local-regional recurrence and distant metastasis. In MeNET, Ki-67-positive cells are usually less than 10%; however, invasive MeNET with low Ki-67 abundance may also become cancerous. In the present case, the Ki-67 proliferation index was less than 5% in primary MeNET, but the tumor recurred 3 times over 9 years and eventually metastasized to the brain with an increased Ki-67 index (10%). In accord with this, a case of MeNET metastasized to the liver (12) showed a much higher Ki-67 index in metastatic brain tumors (6%) compared with the primary tumors (2%).

To further investigate biomarkers related to the risk of MeNET metastasis and recurrence, ATRX, c-Myc, ANO1, and p53 were detected by immunohistochemistry in this case, among which ATRX was observed in both primary and metastatic tumor tissues. It has been reported that ATRX mutation could promote tumor progression (21). ATRX gene changes were associated with abnormal alternative telomere lengthening and poor prognosis in pancreatic non-functioning neuroendocrine tumors (22). This suggests that ATRX may be positively associated with tumor stage and metastasis, reduced relapse-free survival time, and tumor-associated survival time. Above all, increases in Ki67 index and ATRX are potential biomarkers for metastatic MeNET. Characteristics of the present case indicate that CK, SYN, increased Ki67 index, and ATRX may be potential biomarkers of invasive MeNET. This is greatly important for the diagnosis and prevention of invasive MeNET.

The time between initial diagnosis of primary and metastatic MeNET has ranged from 13 months to 44 years (10). Follow-ups after initial surgery have included otoscopy, second-look surgery, and radiology. The optimal follow-up schedule after initial surgery is undecided. Nonetheless, close follow-up is essential for early detection, prevention, and treatment of potential recurrences and metastases. Furthermore, the benefit of radiotherapy and chemotherapy requires systematic evaluation.

## Conclusion

This article reported the clinical manifestations and management of a MeNET case. The patient has survived for more than 10 years after 4 operations. Surgery, radiotherapy, and chemotherapy can be effective treatments for MeNET with brain metastasis, but this and the multi-recurrences greatly affect the patient’s quality of life. Therefore, close following and exploration for recurrence-related biomarkers and the distinctive features of metastases are of great importance in MeNET. Reliable predictive factors should be investigated to identify patients who are at high risk of local recurrence and distant metastasis.

## Data availability statement

The raw data supporting the conclusions of this article will be made available by the authors, with due reservation.

## Ethics statement

Written informed consent was obtained from the participant’s legal guardian or next of kin for the publication of any potentially identifiable images or data included in this article.

## Author contributions

YS: Writing – review & editing, Writing – original draft, Methodology, Formal analysis, Data curation. YZ: Writing – original draft, Methodology, Data curation. DC: Writing – original draft, Investigation, Data curation. WZ: Writing – review & editing, Writing – original draft, Supervision, Resources, Funding acquisition, Conceptualization. ZY: Writing – review & editing, Supervision, Software, Resources, Funding acquisition, Conceptualization.
